# Laboratory Measurements of the Wave‐Induced Motion of Plastic Particles: Influence of Wave Period, Plastic Size and Plastic Density

**DOI:** 10.1029/2020JC016294

**Published:** 2020-12-16

**Authors:** José M. Alsina, Cleo E. Jongedijk, Erik van Sebille

**Affiliations:** ^1^ Laboratory of Maritime Engineering Department of Civil and Environmental Engineering Universitat Politècnica de Catalunya Barcelona Spain; ^2^ Department of Civil and Environmental Engineering Imperial College London London UK; ^3^ Institute for Marine and Atmospheric Research Utrecht University Utrecht The Netherlands

**Keywords:** coastal waves, marine plastic, wave flume experiment

## Abstract

The transport of plastic particles from inland sources to the oceans garbage patches occurs trough coastal regions where the transport processes depend highly on wave‐induced motions. In this study, experimental measurements of the plastic particles wave‐induced Lagrangian drift in intermediate water depth are presented investigating the influence of the wave conditions, particle size and density on the motion of relatively large plastic particles. A large influence of the particle density is observed causing particles to float or sink for relative densities lower and larger than water respectively. The measured net drift of the floating particles correlates well with theoretical solutions for particle Stokes drift, where the net drift is proportional to the square of the wave steepness. Floating particles remain at the free water surface because of buoyancy and no evidence of any other influence of particle inertia on the net drift is observed. Nonfloating particles move close to the bed with lower velocity magnitudes than the floating particles’ motion at the free surface. The drift of nonfloating particles reduces with decreasing wave number, and therefore wave steepness.

## Key Points

- A 16 m wave flume is used to measure wave transport of floating and non‐floating plastic
- Floating plastic transport correlates well with Stokes drift and buoyancy is the only non‐inertial effect
- Non‐floating particles experience shear forces. Transport varies with particle size and density

## 1 Introduction

The amount of plastic in the aquatic environment is rapidly growing, with plastic litter having been reported in almost every marine environment. Understanding plastic dispersion is a key knowledge gap for plastic litter management and cleaning. It is widely assumed that the main source of plastic litter to the oceans is terrestrial and, therefore, the dominant plastic pathway from land sources to the ocean is via coastal regions. However, although there is relatively good knowledge on how the plastic particles move by oceanic currents, it is not clear how the plastic particles are transported by highly nonlinear coastal waves (see van Sebille et al., 2020, for a recent review on the physical plastic transport). Indeed, most of the existing global numerical models predicting plastic fluxes do not explicitly consider the plastic motion in coastal regions including the potential plastic beaching (i.e., plastic being washed ashore) (Neumann et al., 2014).

The hydrodynamics controlling the motion of plastic in intermediate and shallow water is dominated by nonlinear gravity waves propagating toward the shoreline (wave motions and wave‐induced currents). A particle floating on the free surface of a periodic gravity wave experiences a net drift in the direction of wave propagation termed Stokes drift (Stokes, 1847). The Stokes drift velocity is explained by the difference between the average Lagrangian velocity experienced by the particle and the average Eulerian flow velocity of the fluid (Longuet–Higgins, 1953; van den Bremer & Breivik, 2017). The Stokes drift is a second order velocity (Longuet–Higgins, 1953) of smaller magnitude than the magnitude of the wave orbital motion but, due to the persistent wave action, its influence in global ocean circulation can be significant (McWilliams & Restrepo, 1999) and it has been shown to be an important process in the transport of floating particles toward the shoreline and polar regions (Fraser et al., 2018; Onink et al, 2019).

During their propagation from deep water to the shoreline, surface gravity waves experience changes in their shape due to the seabed influence, evolving from an almost symmetrical wave profile in deep water to a shape with sharp crests and broad, flat troughs in coastal waters. As the Stokes drift depends on the shape of the waves (it is proportional to the square of their steepness), this increase in wave steepness in coastal water therefore increases the magnitude of the Stokes drift. For intermediate/shallow water waves (*kh *<* *3, where *k* is the wave number and *h* the water depth), it is assumed that the total horizontal depth integrated mass transport is zero and the net positive transport associated with the Stokes drift at the free water surface is accompanied by an opposing Eulerian return flow at depth (Longuet–Higgins, 1953). Within the surf zone, wave radiation stress leads to a build‐up of fluid near the shoreline (set‐up) which, in turn, generates a pressure gradient driving an offshore flow near the bed (mean return flow or undertow). The undertow is driven by the local vertical difference between the radiation stress and the set‐up pressure gradient (Svendsen, 1984). As a result, the density of the plastic particles is important in the coastal transport where positively buoyant plastic particles on the free surface experience a wave‐induced net drift onshore whereas neutrally buoyant particles in the water column experience a seaward transport as a result of the return flow (Isobe et al., 2014; Shanks et al., 2015). If plastic particles remain very close to the bottom, within the wave boundary layer, their motion resembles lightweight, noncohesive sediment and the understanding of its motion benefits from the large amount of studies on sediment dynamics (i.e., Nielsen, 1992).

Within the wave boundary layer, the presence of the bottom wall and friction affect the motion of plastic particles. In addition to the purely oscillatory wave motions, waves can also induce net currents in the wave boundary layer (also referred to as boundary layer drift). Two competing generation mechanisms have been identified to determine the boundary layer drift (Kranenburg et al., 2012; Scandura, 2007): an onshore drift resulting from the horizontal nonuniformity of the velocity field under progressive free surface waves (also called “progressive wave drift”), and an offshore drift related to the nonlinearity of the wave shape (“wave shape drift”). The “progressive wave drift” occurs because the presence of the bed modifies the phases of the horizontal and vertical orbital wave velocities, which induces a wave‐averaged downward transport of momentum that drives an onshore net current in the boundary layer (Longuet–Higgins, 1953; Johns, 1970). This onshore boundary layer drift contribution is in opposition to the net current that will be generated in a turbulent bottom boundary layer by a velocity‐skewed or acceleration‐skewed oscillation. The wave shape drift is due to the different characteristics of the time‐dependent turbulence during the on and offshore phase of the wave, introducing a nonzero wave‐averaged turbulent shear stress (Scandura, 2007; Trowbridge & Madsen, 1984). Several studies have indicated an increasing importance of the wave shape drift as *kh* decreases (Kranenburg et al., 2012; Scandura, 2007; Trowbridge & Madsen, 1984) and also a dependence of the boundary layer drift with bottom roughness (Kranenburg et al., 2012).

The wave‐induced particle drift has been studied experimentally in different works (i.e., Calvert et al., 2019; Grue & Koolas, 2017; Lenain et al., 2019; Paprota et al, 2016; van den Bremer et al., 2019) although there is still some confusion about the experimental measurement of the net wave‐induced drift at the interior of the fluid (Monismith et al., 2007; van den Bremer & Breivik, 2017). In a closed tank, the Stokes drift of a periodic wave train must be accompanied by a Eulerian return current so that the steady‐state depth‐integrated Lagrangian drift is zero. In the absence of vorticity and viscous effects, this Eulerian return current should be independent of depth. Longuet–Higgins (1953) presented a “convection solution” for large values of a/δ (where *a* is the wave amplitude and *δ* the wave boundary layer thickness) in which vorticity is transported with the mass‐transport velocity from the wavemaker or the other flume end where vorticity can be generated. Most experimental conditions are in this range of a/δ values. According to Longuet–Higgins (1953), the wave paddle starts generating waves on still water conditions and vorticity is advected through the wave flume. Longuet–Higgins (1953) also pointed out that the convection solution may not be stable. In several studies (Monismith et al., 2007; Swan, 1990), the vorticity convection has been shown to play a role and the averaged Lagrangian wave drift velocity (where the Lagrangian velocity is equal to the Eulerian velocity plus the irrotational Stokes drift) has been found to be zero across all of the water column. This implies that a Eulerian mean velocity locally cancels the Stokes drift in those experiments. To overcome these potential problems, van den Bremer et al. (2019), instead of using constant wave trains, measured Lagrangian trajectories using deep water wave groups, allowing the calculation of the Eulerian return flow driven by radiation stress gradients. van den Bremer et al. (2019) showed that Lagrangian displacements in both horizontal and vertical directions within the group can be predicted using inviscid Stokes theory. Calvert et al. (2019) extended this work to analytically solve the Eulerian and Stokes drift for various values of *kh* and wave group length to water depth ratios showing good match with experimental measurements.

However, many other experimental conditions reported in the literature do not agree with the vorticity convection leading to a vanishing Lagrangian drift velocity (i.e., Grue & Koolas, 2017; Hwung & Lin, 1990; Paprota et al, 2016, among others). Paprota et al. (2016) computed the wave drift of relatively short wave trains using particle image velocimetry and the computed trajectories matched well with the irrotational Stokes drift and a constant‐depth Eulerian current velocity. Similarly, Grue and Koolas (2017) performed laboratory measurements of wave‐induced net drift in intermediate water, finding good agreement between the nonlinear irrotational Stokes drift solution with a superimposed return flow within the water column. Despite the good agreement in the interior of the fluid, Grue and Koolas (2017) observed important additional streaming at the free surface and at the bottom boundary layer.

Deike et al. (2017), Lenain et al. (2019), and Pizzo et al. (2019) performed direct numerical simulations (DNS) and laboratory experiments with floating particles, observing drift under breaking and nonbreaking focusing deep‐water wave packets. Deike et al. (2017) using DNS simulations found that the classical Stokes drift described well the numerical simulations for nonbreaking waves. However, for breaking focusing wave packets, they observed a net drift at the surface significantly larger than the classical Stokes drift. Consequently, Pizzo et al. (2019) using the same model as in Deike et al. (2017) quantified the total wave breaking induced transport in the open sea surface to be up to 30% of the predicted Stokes drift. Finally, Lenain et al. (2019) using laboratory measurements found good agreement with the DNS numerical simulations. However, the above‐mentioned works study the transport of passive particles in deep‐water wave conditions with and without wave breaking induced by wave group focusing.

Most studies on wave‐induced drift consider passive particles. Whether plastic particles of all shapes, densities and sizes are transported at the same speed under similar wave conditions remains an open question. Small particles or particles with similar density to the surrounding fluid are expected to move with the Lagrangian flow whereas larger objects or objects with a different density than the surrounding fluid experience forces like drag, inertia, added mass and buoyancy that might modify the particle's motion (Maxey & Riley, 1983). Most studies integrate both effects (density and size) by using the nondimensional Stokes number (*St*). For weakly inertial particles ($S_{t}\ll 1$), theoretical studies have shown that positively buoyant particles (upward settling velocity) move with a slightly faster velocity than the Stokes drift of the surrounding fluid due to the particle inertia whereas negatively buoyant particles (downward settling velocity) move slower than the Stokes drift (Eames, 2008; Santamaria et al., 2013). However, to the authors’ best knowledge, this type of theoretical analysis has never been tested experimentally. DiBenedetto et al. (2019) performed experimental studies analyzing the wave‐induced motion of plastic particles with different shapes finding that the particle shape affected the particles alignment with the flow but they found no evidence of variations in the net particle movement.

The present study investigates the wave‐induced motion of inertial particles in intermediate water depth (0.3* *<* kh *<* *3) using laboratory wave conditions. Spherical particles with different density and size have been used in wave conditions with varying wave steepness. Section 2 introduces the experimental set‐up, wave conditions, the optical measuring system and particle characteristics; followed by the results presented in terms of Lagrangian motion in Section 3. Finally, discussion and conclusions are presented in Sections 4 and 5 respectively.

## 2 Experimental Setup and Data Analysis

### 2.1 Experimental Setup

**Figure 1:**
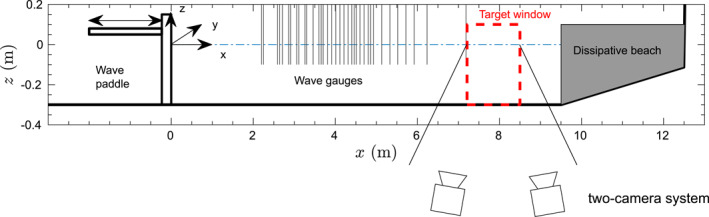
Layout of wave flume, experimental setup, and instrumentation.

The experimental measurements were carried out in a medium scale wave flume, iCIEM, at the Universitat Politècnica de Catalunya. This wave flume has a length of 16 m, a width of 0.40 m and a working water depth of *d *=* *0.30 m (see Figure 1). The system coordinate for the flume geometry (*x*, *z*) has a horizontal coordinate origin at the wave paddle in still water condition and positive in onshore direction whereas the vertical coordinate has its origin at the still water level (SWL) surface and positive upwards (Figure 1). The movement of plastic particles is measured in a planar flume section at a distance of *x *=* *7.5m from the wave paddle. An absorbing section has been built at the wave flume end opposite to the wave paddle. The absorbing beach is composed of a beach section made with timber panels with a slope of 1:15 and starting at *x *=* *9.5 m and *z* = −0.30 m, and ending at *x* = 12.5 and *z* = −0.1 m. The sloping beach section has synthetic foam placed in three compartments separated by grids. The dissipative beach is intended to absorb any incoming wave reducing wave reflection to the minimum, where a detailed reflection analysis will be shown in Section 2.3.

Waves are generated using a piston‐type wave paddle which is numerically controlled by a computer using first order wave generation. The water surface elevation is measured at different cross‐shore locations using a set of seven resistance‐type wave gauges (RWG). Five of these RWGs were placed in a mobile frame that, with repeated experiments, allowed for a large spatial resolution in the surface elevation data set. Two RWG sensors were placed at fixed locations (*x* = 6.24 m and *x* = 7.19 m) just shoreward of the measuring window.

The generated wave conditions correspond to monochromatic (regular) waves with a wave height *H* = 0.06 m measured at *x* = 3.066 m (*x*‐location separated from the wave paddle more than five times the water depth to ensure that the evanescent wave modes near the wave paddle have decayed). The wave period is varied and four different wave steepness conditions, *S* = *ak* where *a* is the wave amplitude (=*H*/2), are studied (see Table 1 for details). According to Table 1, the generated waves propagate in intermediate water depth (i.e., 0.3 < *kh* < 3) and are nonlinear waves that can be represented by a 3rd order Stokes theory (M1 and M2) and a 2nd order Stokes theory (M3 and M4), see Figure 15–7 in Le Méhauté (1976) with the values of *H*/*gT* ^2^ and *h*/*gT* ^2^ indicated in Table 1.

**Table 1:** *Generated Wave Conditions*

Wave condition	*H* (m)	*T* (s)	*S* = *a* $\dot{k}$ (−)	*h* (m)	*kh* (−)	*c* (m/s)	$\frac{H}{gT^{2}}$ (−)	$\frac{h}{gT^{2}}$ (−)
M1	0.060	0.75	0.219	0.30	2.20	1.136	0.0108	0.0544
M2	0.063	1.00	0.144	0.30	1.37	1.373	0.0064	0.0306
M3	0.064	1.50	0.0866	0.30	0.80	1.577	0.0029	0.0136
M4	0.056	2.00	0.0544	0.30	0.58	1.629	0.0014	0.0076

### 2.2 Particle Characteristics

Commercial plastic spheres and spheres fabricated with wax and an additive to control the density have been used to study the influence of size and density in the wave‐induced transport (see Table 2). Their sizes range between 4 and 12 mm and their densities between 760 and 1,340 kg/m^3^ which is within the range of some of the most frequent plastic material densities found in ocean litter (Erni–Cassola et al., 2019). The water density in the flume is assumed to be 1,000 kg/m^3^ obtaining relative plastic particles densities $s_{p}=\frac{\rho_{p}}{\rho_{f}}$ in the range of *s*_*p*_ = 0.760−1.340, that is, positively (*s*_*p*_ < 1) and negatively (*s*_*p*_ > 1) buoyant plastic particles.

**Table 2:** *Plastic Spherical Material Tested Indicating Particle Diameter (d*_*p*_ *), Relative Density (s*_*p*_ *), Settling Velocity (*−*w*_*p*_ *), and Stokes Number (St)*

Material (m)	*d*_*p*_ (mm)	*s*_*p*_ (−)	−*w*_*p*_ (m/s)	*St* (−)
Wax	8.0	0.760	0.132	0.042
Wax with additive 1	8.0	0.910	0.080	0.026
Wax with additive 2	8.0	1.022	−0.039	0.012
Polypropylene (PP)	4.0	0.840	0.074	0.024
	8.0	0.840	0.108	0.034
	12.0	0.840	0.133	0.042
Nylon	4.0	1.100	−0.058	0.019
	8.0	1.100	−0.085	0.027
Polyoxymethylene (POM)	4.0	1.340	−0.110	0.035
	8.0	1.340	−0.158	0.050
	12.0	1.340	−0.194	0.062

*Note*. Note that we plot a negative settling velocity to be consistent with the coordinate axis in Figure 1 where positive velocity indicates upward buoyant particles.

The behavior of the plastic particles in the wave‐induced flow can be assessed by computing the particle Stokes number ($S_{t}$), defined as the ratio between the time scale for the particle to react and the flow time scale. Since we are assuming that the weakly inertial plastic particles are transported with the wave orbital velocity, *S*_*t*_ is computed as,

(1) $$S_{t}=\omega \;\tau_{p},$$

with *ω* the wave angular frequency and $\tau_{p}$ the particle response time. The response time is defined as the relaxation time or constant time in the decay of the particle settling velocity due to drag, $\tau_{p}=w_{p}/g$, where *w*_*p*_ is the particle settling velocity and *g* is the gravitational acceleration. *w*_*p*_ can be obtained as:

(2) $$w_{p}=\sqrt{\frac{4\left(s_{p}-1\right)\;g\;d_{p}}{3\;C_{D}}},$$

where *d*_*p*_ is the particle diameter and *C*_*D*_ is the drag coefficient. This drag coefficient can be expressed as (Fredsøe & Deigaard, 1992):

(3) CD=1.4+36Rep,

where Re_*p*_ is the particle Reynolds number:

(4) Rep=wpdp/ν,

with *ν* is the water kinematic viscosity (=10^−6^ m^2^s^−1^). Many studies on plastic particle transport compute *w*_*p*_ assuming that the drag exerted by the flow on the plastic particles is caused by viscous forces for low values of the particles Reynolds number (Stokes law) (i.e., Eames, 2008; Santamaria et al., 2013). This seems unrealistic in the present experiments where the typical particle Reynolds number ranges between 100 and 3,000. Therefore *w*_*p*_ is obtained solving Equations (2), (3), (4) iteratively. The computed particle settling velocity and Stokes number are displayed in Table 2.

Several studies have analyzed the theoretical influence of particle buoyancy on the wave‐induced particle motion (i.e., Eames, 2008; Santamaria et al., 2013). In those studies a weakly inertial approximation, $S_{t}\ll 1$, is generally considered where the particle response time is much smaller than the wave period $\tau_{p}/T\ll 1$. In the present experimental conditions, *S*_*t*_ is typically lower than 1. Therefore, the tested particles are considered as weakly‐inertial particles (according to *S*_*t*_) with low response times to the wave motion.

In the present experiments, a large influence of the particle relative density (*s*_*p*_) has been found. The experiments start with the particles at still water where the positively buoyant particles stay at the surface whereas the negatively buoyant particles rapidly sink to the flume bottom because of their settling velocity. When the wave motion starts, the floating particles (*s*_*p*_ < 1), released at the surface, move at the water surface whereas the nonfloating particles (*s*_*p*_ > 1), released at the bottom, move parallel to the flume bottom in a sliding or jumping motion as will be explained later in more detail. Some initial tests were performed where nonfloating particles (*s*_*p*_ > 1) were released at the water surface in a developed wave motion but for these specific particles, their buoyancy dominated the motion and they rapidly sunk to the flume bottom before the effect of other inertial processes could be observed.

### 2.3 Analysis of Wave Hydrodynamics

The large spatial resolution in the RWGs allows a detailed characterization of the wavefield. The outgoing wave energy reflected at the absorbing beach is computed to verify the quality of the wavefield as the outgoing wave energy might potentially affect the particle trajectories. A separation technique developed by Padilla and Alsina (2020) (see also Padilla & Alsina (2017) for more details on the application to wave generation) is used, utilizing the whole set of wave gauges. Separation of the ingoing and outgoing waves is performed up to second order components (i.e., *f* and 2 *f*, with *f* the frequency of the primary target component *f* = 1/*T*). More details on the separation technique are illustrated in Appendix A.

**Figure 2:**
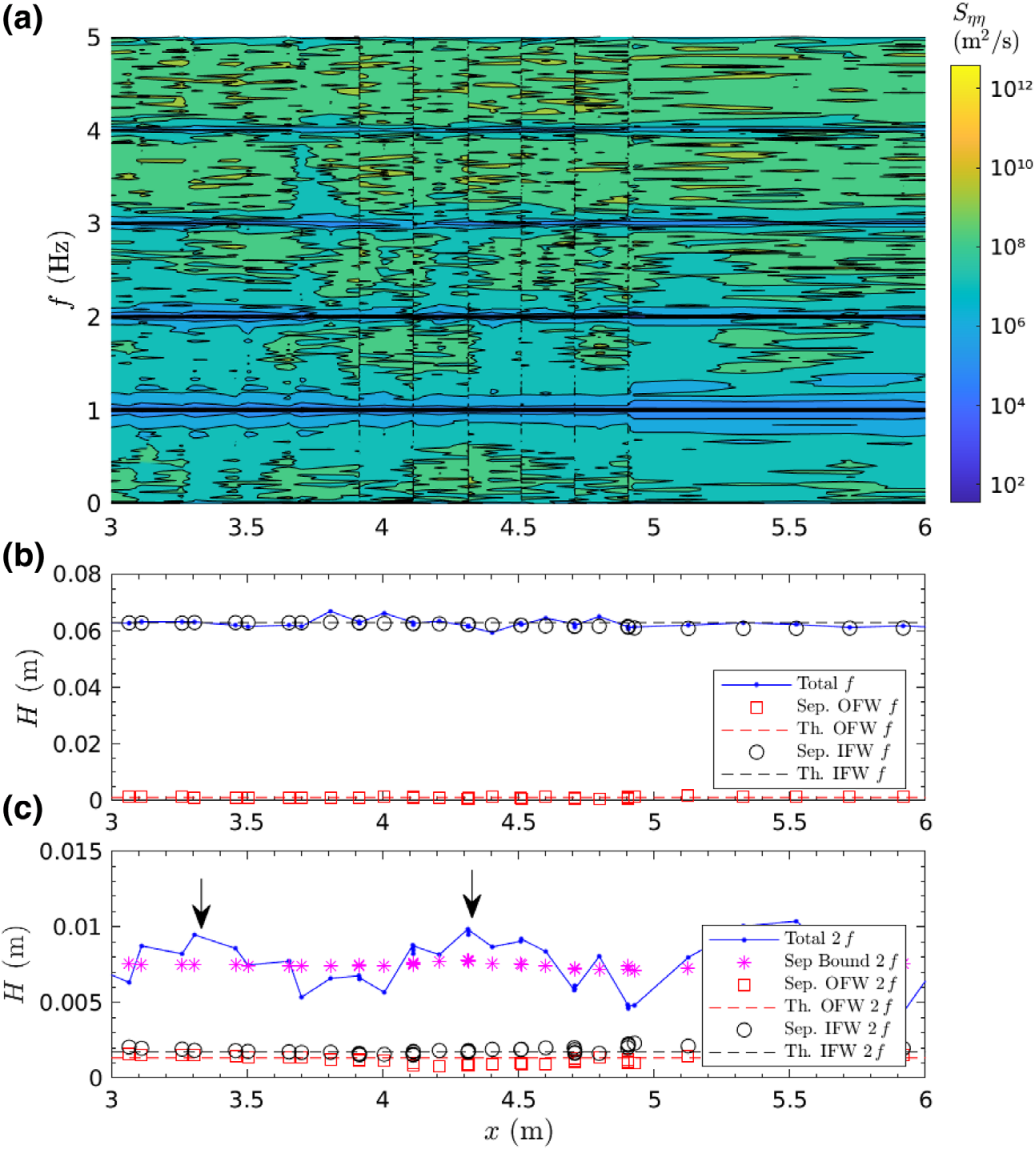
Contour plot of power spectrum density (log‐scale) in *x*‐*f* plane (a); *x‐*distribution of wave heights for main frequency *f* (b) and  2*f* (c) where OFW indicate Outgoing Free Wave, IFW Ingoing Free Wave, Sep. denotes separated components and Th. theoretical computed using linear theory. The vertical arrows indicate the location of computed quasi‐antinodal locations resulting of the ingoing/outgoing separated waves at 2*f* (Padilla & Alsina, 2020).

The use of repeated conditions moving the sensors location allows a good spatial resolution for wave separation. Figure 2 shows an example of the power spectral density distribution in log‐scale for *x* and frequency *f*, and the wave height cross‐shore distribution for different frequency values (*f* and 2*f*) and components (ingoing, outgoing) for wave condition M2. In Figure 2a, the evolution of the primary wave frequency *f* = 1Hz and superharmonics 2*f*, 3*f* is observed. As the bottom is planar, the energy at the different components remain constant, with the primary component *f* being the most energetic component. The separation technique has been performed over *f* and 2*f*. The separation output (Figure 2b) shows a very small reflection of the *f* component (outgoing free wave, OFW, compared to the ingoing free wave, IFW) where most reflection occurs at the higher component 2*f*. At 2*f*, the predominant component is the bound component (bound to *f*, traveling at the phase celerity of *f*) which controls (with other superharmonics) the asymmetry of the generated wave. A free ingoing wave (IFW) in the form of spurious wave is observed at 2 *f* but its energy is very small compared to other components (around 1% of the total energy).

Reflection coefficients are computed as the % of the ingoing amplitude being reflected and obtained at the planar section close to the wave paddle. Reflection coefficients are generally low, and dependent on the wave period. Computed reflection coefficients are of 1.94%, 2.80%, 5.12%, and 6.59% for wave periods 0.75, 1.00, 1.50, and 2.00 s respectively. The outgoing wave amplitude has some influence on the measured trajectories of floating particles in the form of added variability to the computed net Lagrangian motions. For larger wave periods (i.e., *T* = 2.50 s with a reflection coefficient of 14.42%) the computed trajectories start to feel the reflected amplitude showing some deviation from the circular backward trajectory and those data were discarded. For nonfloating particles, the influence of reflection induces a larger variability, as will be illustrated in Section 3. This is attributed to the smaller Lagrangian velocities for nonfloating particles.

Wave height is computed along the different cross‐shore locations of the wave flume from the standard deviation (*σ*) of the measured water surface signal (*η*) as $H=\sqrt{8}\;\sigma_{\eta }$.

### 2.4 Particles Trajectories Analysis

The motion of the plastic particles is recorded using two synchronized IDS UI–31800CP–M–GL video–cameras with a resolution of 5.1 megapixels and shooting at a sampling frequency of 60–80 fps depending on wave conditions. The two video cameras were located at one side of the wave flume facing the measuring window. The measuring window covered an area of roughly 0.925 m (2592 pixels) in the horizontal direction parallel to the wave propagation direction and 0.321 m (648 pixels) in the vertical centered at the water surface for floating particles and close to the flume bottom for nonfloating particles. The pixel resolution is around 0.36 mm per pixel. The use of two synchronized video cameras allows obtaining the three dimensional (3D) particles trajectories by using the stereoscopy concept and epipolar distances (Willneff, 2003). Video images calibration is performed using a three dimensional reference grid placed in the measuring window and at known distances in the flume coordinate system. The 3D calibration grid is recorded with the two cameras at the beginning of each experiment in order to obtain the transformation matrix that will transform image pixel into experimental metric distances. The calibration grid is made using black Lego pieces glued to a timber panel forming a 3D pattern with 8 mm white spheres glued at known *x*, *y*, and *z* locations.

The video cameras are set in two different spatial configurations depending whether the particles move on the water surface or parallel to the flume bottom. The mean distance of each of the cameras with respect to a point located at the center of the measuring window and the bottom of the wave flume at around *x* = 7.79 m, *y* = 0.0 m, and *z* = −0.30 m is indicated in Table 3. When the particles move at the water surface, the cameras are set approximately at the same vertical elevation as the still water level with a mean separation of 1.120 m between the two cameras in the direction of wave propagation. However, when the particles move close to the bed, camera 1 is located at a vertical elevation close to the flume bed and camera 2 is recording the particles motion from the same cross‐shore location but at a higher vertical elevation, which allows for tracking in the *y*‐direction. The illumination system consisted of a high power light‐emitting diode (LED) lamp built in‐house using five lines of high power LED lamps located on a mobile frame on top of the flume walls.

**Table 3:** *Locations of Cameras 1 and 2 with Respect to a Point at the Center of the Measuring Window Located at x* = 7.79 *m, y* = 0 *m, and z* = −0.30 *m*

Camera configuration	Camera	*x*_*c*_ (m)	*y*_*c*_ (m)	*z*_*c*_ (m)
Floating particles with *s*_*p*_ < 1	cam1	0.560	1.450	0.27
	cam2	−0.560	1.450	0.27
Particles moving close to the bed with *s*_*p*_ > 1	cam1	−0.01	1.450	0.750
	cam2	−0.01	1.450	0.120

The video images are recorded and stored using the Norpix StreamPix high speed digital recording software (https://www.norpix.com/products/streampix/streampix.php). The calibration and tracking analysis to obtain the 3D trajectories is performed using the opensource Openptv software (https://www.openptv.net/) (Lthi et al., 2005; Mark et al., 2010) with flowtracks processing software (Meller & Liberzon, 2016).

The Openptv software performs the following tasks (Willneff, 2003): (i) calibration of the camera system, (ii) particles detection, ii. establishment of stereoscopic correspondences determining 3D particle coordinates and (iii) tracking the particles motion in 2D image and 3D object space. The calibration of the two‐camera system is performed using the 3D calibration grid and information of the cameras location with respect to the grid (Willneff, 2003), camera orientation, lens and geometry of the laboratory conditions (each light ray reflected from a particle arriving to the sensor passes three optical media: air, glass and water with different refractive indices). Images are extracted from synchronized video recording. Image preprocessing using high‐pass filtering is performed to reduce nonuniformity in the background illumination, detection of plastic particles in the images is done by a thresholding operator (gray threshold in the image) which localizes particles with subpixel accuracy using a centroid operator. Stereoscopic correspondences are established by correlations (epipolar line) between the two cameras. 2D and 3D particle coordinates are obtained from images using information from the cameras orientation and calibration data (Willneff, 2003).

Trajectories are stored and analyzed to obtain the net particle drift. The Lagrangian drift is evaluated using the methodology proposed by Grue and Koolas (2017) in which the path of the backward time integration is translated in the *x*‐ and *z*‐ direction until it fits with the path of the forward time integration, evaluating the distance function,

(5) $$d=\frac{1}{T_{L}}\int_{t_{0}-T_{L}/2}^{t_{0}+T_{L}/2}\sqrt{{\left(x_{L}\left(t+T_{L}\right)-x_{L}\left(t\right)-x_{0}\right)}^{2}+{\left(z_{L}\left(t+T_{L}\right)-z_{L}\left(t\right)-z_{0}\right)}^{2}}dt,$$

where *x*_*L*_ and *z*_*L*_ denote the Lagrangian horizontal and vertical positions, respectively with respect to the wave flume coordinate system. The Lagrangian period (*T*_*L*_) and the drift distances *x*_0_ and *z*_0_ are obtained minimizing the function *d* in Equation 5. A vector of *T*_*L*_ is generated with a time separation given by the sampling frequency (60–80 Hz) and the minimal function *d* is obtained where *x*_0_ and *z*_0_ are the horizontal and vertical drift given by *T*_*L*_. The starting time *t*_0_ is varied within the measuring time interval. Trajectories are computed over time windows between 12 and 36 s (between 7 and 12 times the wave period) during which the particle trajectories are relatively stable. Trajectories of insufficient time duration <3 *T*_*L*_ are discarded. Some results of Lagrangian drift with very large variability were discarded and treated as outliers. The discarded data are associated to cases with not enough reliable trajectories, particles moving too close to the flume walls (viscous effects) or aggregation of particles with potential influence in particle detection.

The horizontal Lagrangian velocity *U*_*L*_ is obtained as

(6) $$U_{L}=\frac{x_{0}}{T_{L}},$$

where the horizontal drift distance *x*_0_ and the Lagrangian period *T*_*L*_ are obtained from Equation 5 minimizing the *d* function. The average vertical particle coordinate on a wave period is obtained as

(7) $$\bar{z}=\frac{1}{T_{L}}\int_{t_{0}-T_{L}/2}^{t_{0}+T_{L}/2}z_{L}\left(t\right)dt.$$

The trajectory analysis to obtain the particles horizontal and vertical drift distance and drift horizontal velocity (Equation 5–7) is performed on a time window of around 24 s of duration. The average number of Lagrangian wave cycles *T*_*L*_ used to compute the Lagrangiand drift is of 60 cycles for floating particles (minimum of 8 and maximum of 214) and 123 waves for nonfloating particles (minimum of 12 and maximum of 509). The cameras start to record at around 3–5 s after the wave paddle starts moving and are stopped 36 s after the recording start. The initial 12–15 s (depending on wave period) are discarded as the particles might be influenced by the wave paddle ramp up and the horizontal drift might not be stationary. Therefore, the measuring time interval since the wave paddle starts is in the range of 15–41 s. During the selected measuring interval, the trajectories were observed to be nearly stationary, given by a periodic motion and a steady drift. Longuet–Higgins (1953) predicted that the vorticity is “convected” along the flume with the wave mass transport for large values of a/δ. For the present experimental condition, the larger theoretical drift velocity is obtained for wave condition M1, resulting in maximum Stokes velocity at the surface of 0.072 m/s (first term in Equation 8). As the trajectories measuring occurs in a cross‐shore distance of *x* > 7.0 m from the wave paddle, the horizontal conduction of vorticity might affect the mean velocity when time >90 s in the fastest scenario (M1) (Longuet–Higgins, 1953). Therefore, in the present measuring time interval, the convection of vorticity should not affect the measurements.

With two cameras, 3D (*x*,*y*,*z*) trajectories can be obtained. In practice we are interested in the 2D particle motion in cross‐shore (*x*) and vertical (*z*) direction (see Figure 1). To check whether there was no motion in the alongshore (*y*) direction, the 3D results have been analyzed finding minimal motion in the alongshore direction, of the order of mm, and has negligible contribution to the net particle motion and within the variability of the net motion observed between different particles. Therefore, as also the quality and time duration of 2D measured Lagrangian trajectories were larger; the particle transport analysis is presented on the basis of 2D particles motion.

## 3 Results

**Figure 3:**
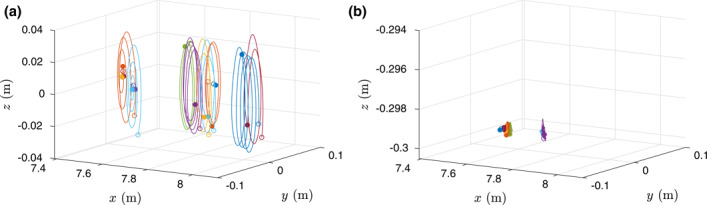
Example of three dimensional plot of Lagrangian trajectories for floating particles (a) and nonfloating particles (b). (a) Corresponds to a wave condition M2 and particles of *d*_*p*_ = 8 mm and *s*_*p*_ = 0.840 whereas (b) correspond to wave M1 and particles of *d*_*p*_ = 4 mm and *s*_*p*_ = 1.10. Note the different *z* scale.

The analysis of the particles Lagrangian drift is separated in floating particles (*s*_*p*_ > 1) moving at the surface, and nonfloating particles (*s*_*p*_ < 1) that move close to the flume bottom. Note that the wave action starts when the particles are already floating on the surface or at rest at the flume bottom. Figure 3 shows an example of 3D particles trajectories with *s*_*p*_ values lower than 1 (Figure 3a) and higher than 1 (Figure 3b). Figure 3 shows that particles moving close to the flume bottom have a shorter trajectory than the orbital motion of the floating particles at the surface. Since the nonfloating particle motion depends on the wave height and wave length ratio to water depth, the nonfloating particles might move faster in shallower water depths than in present conditions.

**Figure 4:**
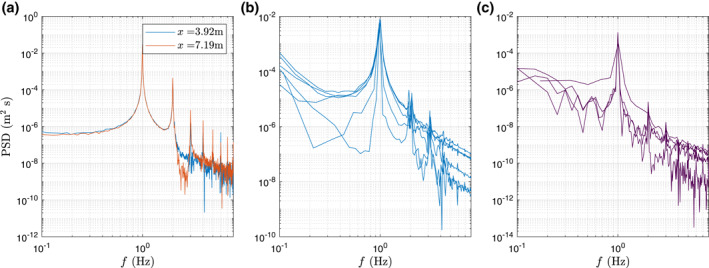
Computed power spectral density of water surface elevation at two different cross‐shore locations for wave condition M2 (a), *x‐*component floating particles trajectory, *d*_*p*_ = 4 mm and *s*_*p*_ = 0.84 (b) and *x*‐component of nonfloating particle trajectory, *d*_*p*_ = 12 mm and *s*_*p*_ = 1.34 (c).

The power spectral density (PSD) of the measured water surface elevation and of the measured *x*‐component of the particle trajectories are displayed in Figure 4 for wave condition M2. Two wave gauge sensor location are displayed in Figure 4a (*x* = 3.92 and *x* = 7.19m) and the power spectral density of the floating (Figure 4b with *d*_*p*_ = 4 mm and *s*_*p*_ = 0.84) and nonfloating particles (Figure 4c with *d*_*p*_ = 12 mm and *s*_*p*_ = 1.34) are illustrated. The energy of the water surface elevation and horizontal particle trajectories is dominated by the primary wave harmonic 1/*T* with influence of bound superharmonics 2/*T* and 3/*T*. However, there is no appreciable presence of energy at longer periods in the water surface elevation signal. The PSD of the water surface elevation is computed using a time series of 360 s whereas the PSD of particles trajectories (*x*‐component) is computed using the variable time window of the tracked trajectories (between 10 and 20 s). The different time window in the particle trajectories explains the differences in the PSD. The net drift is a zero frequency component and it does not appear in the PSD figures.

**Figure 5:**
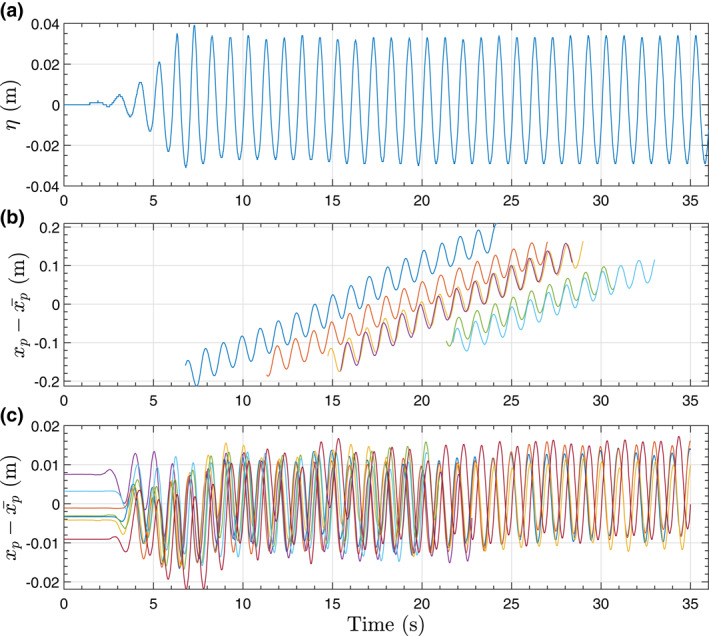
Measured water surface elevation at *x* = 7.19 m for wave condition M1 (a) and time series of *x*‐component particle trajectories minus the mean value ($x_{p}-\bar{x_{p}}$) for floating (*d*_*p*_ = 4 mm and *s*_*p*_ = 0.84) (b) and nonfloating particles (*d*_*p*_ = 12 mm and *s*_*p*_ = 1.34) (c).

Although the particles motion is induced by the wave frequency components, a variability in the computed net Lagrangian drift using Equations 5 and 6 was observed. This variability is significant for nonfloating particles as the net Lagrangian drift is much smaller than for nonfloating particles. The variability occurs at the primary wave components and superharmonics as suggested in Figure 4 and it is attributed to the superposition of ingoing and outgoing components as illustrated in Figure 5. In Figure 5a, variability in the water surface elevation is observed as a result of the superposition of ingoing and outgoing components at *f* and 2*f*. This variability based on wave trains superposition can be explained up to second order by the separation analysis presented in Section 2.3. The floating particle trajectories are relatively stable as the particles move largely with the ingoing primary components. However, for the nonfloating particles, the particles motion is smaller and the wave superposition induces a large variability over the net drift.

### 3.1 Lagrangian Drift of Floating Particles

**Figure 6:**
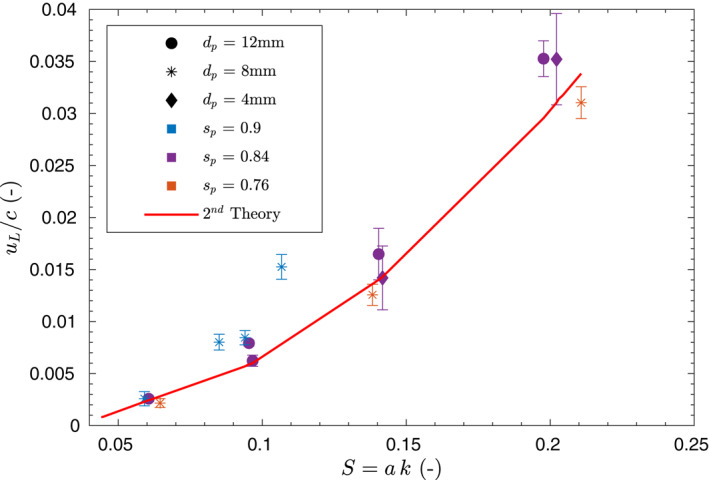
Nondimensional Lagrangian drift measured over different steepness (*S*) for floating (*s*_*p*_ < 1) particles with varying size and densities. Solid line represents the theoretical second order solution. Note that *a* and *h* are kept constant and only the wave period is varied.

The computed Lagrangian drift using Equation 6, made nondimensional with the wave celerity *c*, is shown in Figure 6 for floating particles with *s*_*p*_ < 1. The Lagrangian drift of the particles is averaged over the number of wave cycles with period *T*_*L*_ and averaged over the total number of computed trajectories for each wave condition. The mean and standard deviation are shown in Figure 6 as error bars. The variability in the Lagrangian drift over different trajectories is generally below 30% of the mean obtained value. Note that the Lagrangian drift is a quantity resulting from cumulative contribution of larger orbital velocities where a small variability in the magnitude of the orbital velocity can result in a relatively large contribution to the net drift. Other authors (Grue & Koolas, 2017; Lenain et al., 2019) have also reported similar variability in laboratory obtained Lagrangian drift. The reasons for the obtained variability can be attributed mostly to wave generation and influence of the ingoing and outgoing components. Other affecting variables are time resolution in the drift velocity computation minimizing the function *d* in Equation 5, video camera resolution, image calibration and proximity of the particles to the glass flume wall and viscous effects. Missing data in Figure 6 corresponds to a lack of trajectories larger than 3*T*.

The second order Lagrangian drift solution is computed as (Longuet–Higgins, 1953),

(8) $$u_{L2nd}=\frac{a^{2}\;\omega \;k\;\cosh\left(2k\left(\bar{z}+h\right)\right)}{2\sinh^{2}\;\left(kh\right)}-\frac{a^{2}\omega }{2h}\coth\left(kh\right),$$

where *k* is the wave number, *h* is the water depth, *ω* is the angular frequency, and *a* is the wave amplitude. The first term in Equation 8 is the classical Stokes solution (Stokes, 1847) whereas the second term is the return flow that ensures a net zero depth integrated mean Lagrangian flow (Longuet–Higgins, 1953). Second order Lagrangian drift obtained from Equation 8 is also displayed in Figure 6 for reference.

Figure 6 shows that the obtained Lagrangian particle drift for floating particles closely follows the 2nd order theory independently of size and density for the given experimental conditions. Only one data point (*s*_*p*_ = 0.9, *d*_*p*_ = 8 mm and M1) seems to deviate from the second order solution in nondimensional form. Note that in Figure 6 the wave steepness is varied as the wave number (wave period) is varied in the experimental conditions while *a* and *h* remain constant (Table 1). The relationship between *u*_*L*_/*c* and the wave steepness *S* derived from Equation 8 is dependent of *S* ^2^ and *kh* for the present experimental conditions.

Floating particles with different densities and sizes equally follow the theoretical solution for the Lagrangian drift expressed in Equation 8 and besides buoyancy, contribution of other inertial particle effects such as added mass and drag are not observed.

### 3.2 Lagrangian Drift of Nonfloating Particles

**Figure 7:**
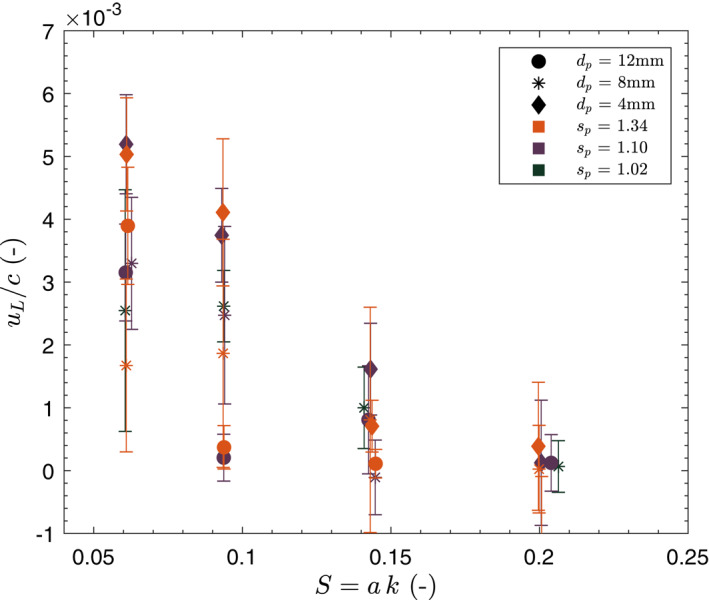
Nondimensional Lagrangian drift measured over different steepness (*S*) for nonfloating (*s*_*p*_ > 1) particles with varying size and densities. Mean values and error bars are plotted showing trajectories variability.

As illustrated in Figure 3, when the particle density is larger than water, the plastic particles move parallel to the bed forced by the wave action. In this case, the wave‐induced force on the particles is of different nature than the direct wave action on floating particles. For plastic particles moving close to the bed, the wave‐induced boundary layer processes play an important role. The nondimensional Lagrangian drift *u*_*L*_/*c* is displayed against steepness *S* for nonfloating (*s*_*p*_ > 1) particles in Figure 7 with error bars indicating standard deviation. The variability in the mean motion between trajectories is significantly larger than for floating particles which is attributed to the influence of wave reflection on the particles trajectories. Note also that the nonfloating particle drift is of smaller magnitude than the Stokes drift. Nevertheless, there is a clear dependency of the Lagrangian velocity *u*_*L*_ on the wave steepness where, as shown in Figure 7, a small steepness reduces the drift of the particles.

For a nonfloating particle, Figure 7 suggests a trend of particle properties (size and density) affecting the particle drift. The drift tends to reduce for increasing particle density and size. However, this trend is not conclusive due to the large variability in particle drift.

**Figure 8:**
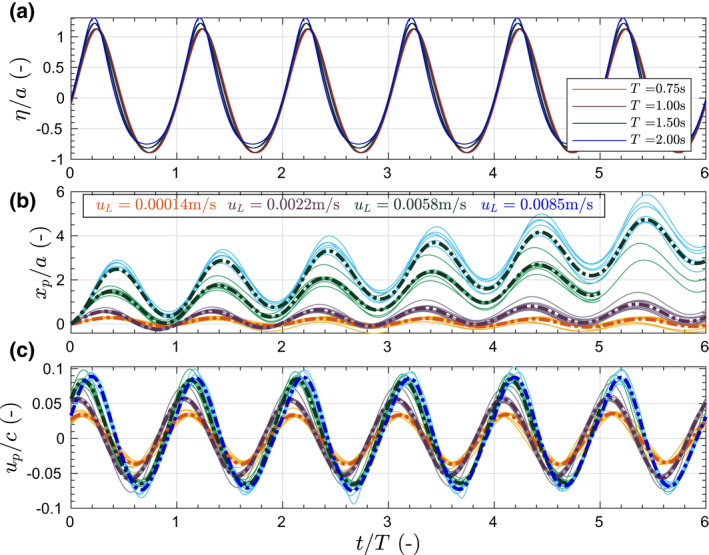
Nondimensional time evolution of water surface elevation (*η*/*a*), horizontal particle displacement (*x*_*p*_/*a*) and horizontal particle velocity (*u*_*p*_/*c*) for different wave periods *T* = 0.75, 1.00, 1.50, and 2.00 s where the wave steepness is respectively *S* = 0.20, 0.14, 0.09, and 0.06 respectively. *d*_*p*_ = 4 mm and *s*_*p*_ = 1.10 for all cases. Both individual particles and mean values are displayed.

The isolated influence of wave period (for a constant wave height), is illustrated in Figure 8. The nondimensional water surface elevation, particle position and particle velocity are illustrated for cases with same particle size and density but varying the wave period (Figure 8). The water surface elevation is obtained from the measured water surface elevation signal propagated from the last RWG to each of the initial particle position.

It is noticeable that the drift of nonfloating particles increases as the wave period increases, contrary to floating particles. The wave orbital velocity amplitude at the bottom, given by $u_{bm}=\left(H\;\pi /T\right)/\sinh\left(kh\right)$ using linear theory, increases with the wave period (as *kh* decreases) which is reflected in Figure 8c as the particles velocity amplitude increases with the wave period. Moreover, although the short waves are steeper in absolute terms (shorter wave periods but constant wave height), in nondimensional form the larger wave period conditions are in shallower water and more asymmetric waves with sharper wave crest and flatter wave troughs (as reflected in Figure 8a). Computed values of wave skewness (horizontal wave asymmetry) indeed increase as the wave period increases with skewness values of 0.25, 0.26, 0.43, and 0.57 for wave periods 0.75, 1.0, 1.50, and 2.0 s, respectively. Close to the bottom, where the shear stress is a function of the square of *u*_*bm*_, this asymmetry results in larger shear force exerted on the particles during the forward motion wave phase (wave crest) than during the backward wave motion (wave trough). Note in Figure 8 the slight phase lag between the particle velocity and the water surface elevation as well with a variable phase lag for different wave periods. Phase‐lags between water surface elevation and particle motion are −46.39°, −60.85°, −31.81°, and −7.59° for wave periods 0.75, 1.0, 1.50 and 2.0 s, respectively. Overall, a trend of decreasing phase lag with increasing wave period is observed with the exception of the wave condition with *T* = 0.75 s.

## 4 Discussion

The wave‐induced motion of floating and nonfloating plastic particles in intermediate water depth has been investigated experimentally. The present experiments mimic the dynamics of coastal nonbreaking waves where the wave‐induced motion has been isolated from other influences (wind and currents). The wave steepness has been varied testing different wave periods keeping the wave energy constant. It is commonly assumed that a large part of the plastic litter enters the marine environment via river discharge where the residence time of plastic particles in intermediate and shallow water (the coastal zone) is largely unknown. Previous studies on plastic particles distribution in coastal regions and comparison with oceanic plastic particles are scarce and often assume empirical formulations for the particles beaching probability.

The present experimental study shows that outside the surf zone, in the shoaling region where wave steepness and asymmetry increases, floating particles move onshore due to the action of Stokes drift. Floating particles move with the Stokes drift velocity with no influence of the particle size and density beyond the buoyancy effect that keep the particles within the free water surface. Floating particles with different size and density move with the same Stokes drift, the difference between those motions is less than or equal to the variability between motions of particles with the same size and density. Within the experimental conditions, floating plastic particles do not detach from the free surface showing a large influence of the particle positive buoyancy. This result supports the measurements of Stokes drift in the open ocean using relatively large floating buoys and drifters (i.e., Herbers et al., 2012) as it implies that the potential inertia of drifters has little influence on the Stokes drift.

As floating particles move shoreward with the wave propagation due to the wave‐induced Stokes drift, it seems unlikely that large floating plastic particles leave coastal water. Therefore, in the absence of currents or wind, the fate of large floating plastic bodies (i.e., bottles, packages, porex, plastic cutlery, bags, straws) seems to be to return to the dry beach. This experimental finding is in line with recent studies suggesting large rates of plastic particles accumulation in coastal regions (Lebreton et al., 2019; Olivelli et al, 2020). However, smaller particles (microplastics) might move within the water column with varying drift direction depending on the particle vertical location within the water column. Since the settling velocity decreases for smaller particles, even with a relatively large value of *s*_*p*_ (*s*_*p*_ > 1) particles can still reside in suspension for a long time exposing them to mid‐column flow processes and will move seaward because of the Stokes drift vertical distribution (i.e., Grue & Koolas, 2017; Stokes, 1847). Indeed, Isobe et al. (2014) using information of plastic accumulation in coastal regions of Japan already suggested that large plastic particles (mesoplastics) remain in the coastal region (surf zone) whereas smaller particles (microplastics) move seaward. Experimentally, it is difficult to obtain large plastic particles moving within the water column. Small variations in the particle density have a relatively large influence in the particle settling velocity and particles show a tendency to either settle or staying in the water surface, independently of the experiments starting from still water or with a wave‐induced mixed water before launching the particles. All the tested plastic particles in the present experiments either moved floating in the water surface or close to the bed. However, in real sea conditions, the plastic size distribution is large with a large presence of microplastic (Eriksen et al., 2014). Mixing is also larger due to several environmental processes absent in the laboratory (currents, white‐capping, wave directional spreading, shear, presence of fronts, density driven currents), and plastic density and size changes can occur over large periods of time due to fouling, degradation and fragmentation which are not considered in experimental conditions.

Nonfloating plastic particles move parallel to the bed transported by the wave boundary layer motion. The variability of the nonfloating particles is larger than for floating particles. This is attributed here to the smaller drift velocities and the influence of wave reflection that, although small, produces a large variability in the net observed drift. Trends of different nonfloating particle drift with different particles size and density are suggested. Unlike floating particles, the motion of nonfloating plastic with different size and densities cannot be obtained with a single drift formulation and a more complex formulation is needed (Maxey & Riley, 1983). The wave‐induced motion on large bottom particles has been, indeed, successfully simulated by Voropayev et al. (1998) using a modified Maxey‐Riley formulation. Other potential aspects influencing the drift of nonfloating particles linked to bed friction have not been studied in the present study (i.e., bed roughness and the presence of bedforms). In the present experiments, for the given wave energy all plastic particles move, reflecting that the initiation of motion threshold (Shields, 1933) is exceeded for all experimental combination of variables. Bed roughness and plastic burial will also affect the initiation of motion threshold.

Several authors have modeled the boundary layer drift considering the different contributions to the net drift (i.e., “progressive wave drift,” “wave‐shape drift”) (Kranenburg et al., 2012; Scandura, 2007, among others). In those boundary layer drift models, the drift velocity generally increases with *kh*. For example, Kranenburg et al. (2012) parameterized the net boundary layer velocity at the top of a rough turbulent boundary layer as:

(9) $$\frac{U_{0}}{c}=0.345+0.7{\left(\frac{A}{k_{N}}\right)}^{-0.9}-\frac{0.25}{\sinh^{2}\left(kh\right)}$$

where *U*_0_ is the net velocity at the top of the boundary layer, *A* is the wave velocity amplitude and *k*_*N*_ is the Nikuradse roughness height. For a constant *A*/*k*_*N*_ value, the net velocity at the boundary layer increases with *kh*, (increases with *S* = *ak* as *a* and *h* are constant). This increase in the boundary layer drift with *kh* has been reported by several authors (Kranenburg et al., 2012; Scandura, 2007; Trowbridge & Madsen, 1984) and it is in opposition to the observed movement of nonfloating particles in the present experiments (decreasing with *k*), suggesting a relatively larger influence of the bottom orbital velocity (bottom orbital velocity also decreases with *k*) for the given experimental conditions. The influence of the boundary layer drift on smaller particles or smaller density nonfloating particles than experienced here is open to further investigation.

## 5 Conclusion

The net drift of plastic particles has been measured in laboratory conditions for periodic waves of different wave steepness and for a varied range of plastic sizes and densities. The motion of the particles is primarily influenced by the particle density, and whether the relative particle density is lower or larger than unity. For floating (*s*_*p*_ < 1) particles, the net motion is induced by wave orbital motion only and particles follow Stokes drift theory. Nonfloating (*s*_*p*_ > 1) plastic particle motion occurs at the bottom of the flume and it is mainly related to the characteristics of the wave orbital motion at the bed. From the obtained measurements it is concluded:

- Floating particles show a positive (shoreward) net drift in accordance to the second order classical Stokes drift theory. This solution and the experimental results show that the particles net drift increases with the wave steepness
- For floating particles, the plastic particles size and density has no influence on the net particle drift apart from the buoyancy that keeps the particles afloat
- The drift of nonfloating particles is shoreward directed (with the wave direction), and its velocity also depends on the wave number, decreasing as the wave number increases
- Nonfloating plastic particles move with the bottom orbital velocity and the net drift of the particles seems to reduce with plastic density and size but trends are nonconclusive and masked by particle motion variability.

The present experiments illustrate the importance of wave‐induced motions for the understanding of plastic litter transport. They suggest a strong tendency for floating and nonfloating particles to drift with the wave propagation direction (toward the shoreline). Further experiments are planned to study the plastic motion within the surf and swash zones.

## Data Availability

The data collected in this work are archived on Zenodo and available at https://doi.org/10.5281/zenodo.3742183.

## Appendix A Wave Separation and Computation of Ingoing and Outgoing Wave Components

### A.1

The computation of ingoing and outgoing wave components to obtain the reflection coefficients presented in Section 2.3 is performed using a wave separation technique as presented in Padilla and Alsina (2020) and resumed here. For a certain wave frequency, *f*, the water surface elevation, η, measured at the different cross‐shore locations in the wave flume, *x*, is formed by the combination of *n* wave trains depending of their propagation nature (i.e., ingoing, outgoing, free or bound wave trains),

(A.1) $$\eta \left(x,t\right)=ℜ\left\{[\overset{n}{\underset{j=1}{\sum }}Z^{j}\left(x\right)]e^{2\pi ft}\right\},$$

where *Z* ^*j*^ is the complex amplitude for each wave train *j*. The wave separation procedure consists in obtaining *Z* ^*j*^ for each existing wave component knowing the wave propagation characteristics between adjacent wave measuring locations within an array of wave gauges consisting of *P* gauges (*p* = 1, … *P*). The wave separation at the centered reference location *x*_*r*_ in the wave gauge array *x*_*p*_ is computed by solving the linear system,

(A.2) $$Z_{p}=\overset{n}{\underset{j=1}{\sum }}\left(Q_{r,p}^{j}Z_{r}^{j}\right),$$

where $Q_{r,p}^{j}=K_{r,p}^{j}e^{i\Phi_{r,p}^{j}}$ represents the propagation coefficients of any wave train from *x*_*p*_ to *x*_*r*_. *K* and Φ are factors that perform the cross‐shore evolution of the amplitude and phase respectively (Padilla and Alsina, 2020). For a planar bed $Q_{r,p}^{j}$ becomes $Q_{r,p}^{j}=e^{ikx_{r}^{p}}$ for free waves and $Q_{r,p}^{j}=e^{i\frac{2\pi f}{c_{B}}x_{r}^{p}}$ for bound waves, where $x_{r}^{p}$ is the cross‐shore distance from *r* to *p* and *c*_*B*_ is the bound wave phase velocity (Padilla and Alsina, 2020). For the general case of a wave train formed by the superposition of an ingoing free wave (IFW), ingoing bound wave (IBW) and outgoing free wave (OFW), the separation is obtained after solving the overdetermined system:

(A.3) $$(\begin{array}{lll} Q_{r,1}^{IBW} & Q_{r,1}^{IFW} & Q_{r,1}^{OFW}\\ Q_{r,2}^{IBW} & Q_{r,2}^{IFW} & Q_{r,2}^{OFW}\\ : & : & :\\ Q_{r,p}^{IBW} & Q_{r,p}^{IFW} & Q_{r,p}^{OFW}\\ : & : & :\\ Q_{r,P}^{IBW} & Q_{r,P}^{IFW} & Q_{r,P}^{OFW} \end{array})\left(\begin{array}{l} Z_{r}^{IBW}\\ Z_{r}^{IFW}\\ Z_{r}^{OFW} \end{array}\right)=\left(\begin{array}{l} Z_{1}\\ Z_{2}\\ :\\ Z_{p}\\ :\\ Z_{P} \end{array}\right),$$
